# The Role of Dissociative Compartmentalization in Difficult-to-Treat Psychotic Phenomena

**DOI:** 10.3389/fpsyg.2021.533884

**Published:** 2021-04-09

**Authors:** Cate Treise, Jesus Perez

**Affiliations:** ^1^CAMEO Early Intervention Services, Cambridgeshire and Peterborough National Health Service Foundation Trust, Cambridge, United Kingdom; ^2^Department of Psychiatry, University of Cambridge, Cambridge, United Kingdom; ^3^Norwich Medical School, Faculty of Medicine and Health Sciences, University of East Anglia, Norwich, United Kingdom

**Keywords:** dissociative, compartmentalization, psychosis, delusion, CBTp

## Introduction

Recommended psychological treatments for psychosis [National Institute of Health and Care Excellence (NICE), 2014] have developed from innovative cognitive models of positive symptoms (Garety et al., 2001) which explain delusions as causal inferences that form in response to affective, perceptual, and cognitive anomalies (Newman-Taylor and Sambrook, 2013). In cognitive behavior therapy for psychosis (CBTp) emphasis is made on symptom modification, optimizing the therapeutic alliance to manage affect and associated stressors, and using cognitive challenging and reality testing to work on attributional and reasoning bias associated with formation and maintenance of delusional beliefs. Recent evolutions such as Freeman's multifactorial model (Freeman, 2016), which does not require an individual to have insight into their symptoms, show promising improvement in CBTp treatment outcomes by focusing intervention on causal factors associated with the formation and maintenance of persecutory delusions (worry, sleep dysfunction, safety behaviors, negative self-beliefs, reasoning bias, and anomalous experience) rather than dismantling the fixed belief itself (Freeman, 2016). However, a significant number of patients with persistent delusions still show little improvement with either pharmacological or psychological treatments (Freeman et al., 2015; Mehl et al., 2015; Skelton et al., 2015; Freeman and Waite, 2017; Treise et al., 2019) and mechanisms underpinning these difficult-to-treat symptoms are not well-understood (Bebbington and Freeman, 2017).

In light of new evidence that different types of psychotic symptoms are strongly associated with dissociation (Longden et al., 2020), that treatment of psychotic symptoms can be enhanced by targeting dissociation (Varese et al., 2020), and growing endorsement of the proposal that some psychotic symptoms may in fact be dissociative in nature (Moskowitz et al., 2009; Longden et al., 2020), a mechanistic hypothesis of the role of dissociative processes in difficult-to-treat delusions may now have wider transdiagnostic value.

## Dissociative Compartmentalization in Difficult-to-Treat Psychotic Phenomena

Dissociation has a well-established presence in psychosis populations (Ross and Keyes, 2004; Moskowitz, 2011; Vogel et al., 2013; Renard et al., 2017; Sun et al., 2018a), and may have a pivotal role in the formation and maintenance of psychotic symptoms (Schäefer et al., 2012; Sun et al., 2018a; Freeman et al., 2019; Treise et al., 2019; Longden et al., 2020). Dissociative symptoms have been found in both longer-term psychotic illness (Ross and Keyes, 2004) and in first episode of psychosis (Sun et al., 2018b), and are mostly associated with positive symptoms, specifically delusions and hallucinations (Spitzer et al., 1997; Ross and Keyes, 2004; Kilcommons and Morrison, 2005; Lysaker and LaRocco, 2008; Schäefer et al., 2012; Vogel et al., 2013; Schroeder et al., 2016; Sun et al., 2018b; Longden et al., 2020; Varese et al., 2020). Moreover, the severity of dissociative phenomena may fluctuate in step with psychotic symptoms, with higher levels of dissociation in the acute illness phase compared to stabilization (Schäefer et al., 2012). Despite evidence supporting a relationship between dissociative and psychosis phenomena, evidence supporting dissociation as a treatment focus for positive symptoms of psychosis (Longden et al., 2020; Varese et al., 2020), and proposals that some psychotic symptoms may be better conceptualized as forms of dissociation (Moskowitz et al., 2009; Longden et al., 2020), the nature of this connection has remained elusive (Sun et al., 2018b; Freeman et al., 2019; Treise et al., 2019). This uncertainty may have contributed to the fact that treatment guidelines [National Institute of Health and Care Excellence (NICE), 2014] have not yet included dissociative processes as targets for routine psychological treatment in populations with psychosis (Newman-Taylor and Sambrook, 2013; Treise et al., 2019).

The American Psychiatric Association description of dissociation as a, “disruption of the usually integrated functions of consciousness, memory, identity, or perception of the environment” (American Psychiatric Association, 1994), reflects the conceptualization of dissociation as a unitary phenomenon varying only in severity (Brown, 2006) which has prevailed in research about dissociation and psychosis. However, conceptualization of dissociation as two distinct processes, proposed as “detachment” and “compartmentalization” by Holmes et al. (2005), is gaining traction in recent explorations (Vogel et al., 2013; Sun et al., 2018b; Treise et al., 2019).

Compartmentalization phenomena are characterized by loss of ability to deliberately control processes or actions that ordinarily an individual would have volitional access to Holmes et al. (2005); an effect that is reversible, but not available to conscious control. Aside from lack of volitional control, the compartmentalized processes operate in a normal manner, influencing cognition, emotion, and actions in a way that the individual experiences as intuitively correct (Holmes et al., 2005), and causing the compartmentalization to be accepted and defended as reality (Treise et al., 2019). Compartmentalization phenomena have been identified in somatoform symptoms such as seizures or paralysis seen in Functional Neurological Disorders, and also in psychoform symptoms such as amnesia, flashbacks, or pseudohallucinations (Brown, 2013). Detachment phenomena are qualitatively different, presenting as an altered state of consciousness in which individuals describe a sense of separation from aspects of everyday life (as in derealization or depersonalization), and are identified as sometimes co-existing with compartmentalization phenomena (Holmes et al., 2005).

A mechanistic hypothesis of the direct role of dissociative compartmentalization was recently proposed in a model of the formation and maintenance of a difficult-to-treat symptom in Delusional Disorder, and may offer clarification transdiagnostically of how and why some phenomena classed as delusions persist. In a clinical setting, Treise et al. (2019) encountered a phenomenon of highly repetitive expression of a fixed belief and associated utterances – for example, “My brain's dying. I can't survive with no sleep. The tablet blocked the nerves from my brain to my body!” (Treise et al., 2019, p. 3)– which appeared episodic in nature, and persisted in the absence of other psychiatric symptoms. The phenomenon described met current DSM-5 criteria for “Delusion”: a false belief based on incorrect inference about external reality despite what almost everyone believes and despite what constitutes incontrovertible and obvious proof or evidence to the contrary (American Psychiatric Association, 2013). The beliefs persisted despite attempts at treatment using medication and psychological approaches, with early attempts at psychotherapy limited by the acceptance and defense of the beliefs, and attempts at therapeutic work with associated factors such as worry (Freeman et al., 2015; Freeman, 2016) limited by apparent difficulty in identifying and working with psychological constructs such as emotions and thoughts (Treise et al., 2019).

Using a hypothesis in which dissociative compartmentalization plays a central role in the maintenance of these beliefs, Treise et al. reconceptualized the phenomenon as a Dissociative Thought-Script (DT-S). The DT-S model is an adaptation of an existing psychological theory of processes known to be associated with functional neurological disorder (FND), the Integrative Cognitive Model (ICM), which represents the central role of compartmentalization (Brown and Reuber, 2016) in the formation and maintenance of unexplained medical symptoms such as psychogenic seizures. Drawing on the ICM (Brown and Reuber, 2016), the DT-S model proposes a reconceptualization of a phenomenon that would usually be classed as a “delusion,” as dissociative in nature and triggered via a threat response to negative affect or associated triggers. Prior expectation drives formation and activation of cognitive-behavioral programmes, described in the ICM as “rogue representations” (Brown and Reuber, 2016), that are dissociated from experience, run as intuitively correct, and are therefore accepted and defended as reality. In the ICM and DT-S models it is proposed that certain dissociative phenomena may form as a threat response when people are unable to process negative emotions and the meaning attached to these, either because they have difficulty representing emotional states symbolically, or because processing negative emotions is too threatening for them (Brown and Reuber, 2016; Treise et al., 2019). The threat response is influenced by expectation based on prior experience, and could originate via any pathway that makes the experience of negative emotions threatening including for example childhood trauma and/or the experience of living with autism spectrum conditions. The role of trauma in formation of such phenomena is possible, but not necessary, and as such the model is different to conceptualizations of trauma-related dissociative phenomena, as seen in PTSD, where peri-traumatic dissociative experiences fail to be integrated and are later re-activated.

A successful psychological intervention for this difficult-to-treat symptom was developed in which emphasis was placed less on delusional content and focused more specifically on dismantling dissociation and underlying affective factors connected with the activation and maintenance of the fixed belief (Treise et al., 2019). As underlying negative affect was attenuated, the beliefs were activated less frequently and with less intensity, episodes of detachment reduced, and thinking became more flexible. This provided a platform for treatment using existing evidence-based psychological therapy.

## Conclusion

As transdiagnostic symptoms, delusions, and the mechanisms supporting them have been identified as a valuable area of investigation to advance clinical interventions (Bebbington and Freeman, 2017). The proposal that dissociative compartmentalization may play a direct role in the formation, maintenance and expression of difficult-to-treat phenomena currently considered as persistent delusions brings together neuropsychiatric and psychological perspectives to reconceptualize certain symptoms classed as psychotic (Treise et al., 2019), and is consistent with growing evidence of the connection between dissociation and psychosis, and emphasis that some symptoms of psychosis may be dissociative in nature (Moskowitz et al., 2009; Longden et al., 2020). Wider investigation of the role of dissociative compartmentalization may present an opportunity to advance our understanding of mechanisms connecting dissociation with psychotic symptoms, and in doing so identify new routes to enhance treatment outcomes in difficult-to-treat symptoms that do not respond well to existing treatment approaches. In the context of a research project supporting treatment of patients with a first episode of psychosis, our intention is to test the viability and clinical outcome of focusing on this particular phenomenon as a transdiagnostic symptom.

## Author Contributions

CT and JP have developed the ideas contained in this paper via work within the same clinical team. The paper was written by CT and further developed and edited together with JP.

## Conflict of Interest

The authors declare that the research was conducted in the absence of any commercial or financial relationships that could be construed as a potential conflict of interest.
